# Enhanced CXCR4 Expression of Human CD8^Low^ T Lymphocytes Is Driven by S1P_4_

**DOI:** 10.3389/fimmu.2021.668884

**Published:** 2021-08-24

**Authors:** Tobias Burkard, Caroline Dreis, Martina Herrero San Juan, Meik Huhn, Andreas Weigert, Josef M. Pfeilschifter, Heinfried H. Radeke

**Affiliations:** ^1^pharmazentrum Frankfurt/ZAFES, Institute of Pharmacology and Toxicology, Hospital of the Goethe University, Frankfurt/Main, Germany; ^2^Institute of Biochemistry I, Faculty of Medicine, Goethe-University, Frankfurt/Main, Germany

**Keywords:** cytotoxic T lymphocyte, tumor immunity, IL-33, chemokines, sphingolipids

## Abstract

Although the human immune response to cancer is naturally potent, it can be severely disrupted as a result of an immunosuppressive tumor microenvironment. Infiltrating regulatory T lymphocytes contribute to this immunosuppression by inhibiting proliferation of cytotoxic CD8^+^ T lymphocytes, which are key to an effective anti-cancer immune response. Other important contributory factors are thought to include metabolic stress caused by the local nutrient deprivation common to many solid tumors. Interleukin-33 (IL-33), an alarmin released in reaction to cell damage, and sphingosine-1-phosphate (S1P) are known to control cell positioning and differentiation of T lymphocytes. In an *in vitro* model of nutrient deprivation, we investigated the influence of IL-33 and S1P receptor 4 (S1P_4_) on the differentiation and migration of human CD8^+^ T lymphocytes. Serum starvation of CD8^+^ T lymphocytes induced a subset of CD8^Low^ and IL-33 receptor-positive (ST2L^+^) cells characterized by enhanced expression of the regulatory T cell markers CD38 and CD39. Both *S1P_1_* and *S1P_4_* were transcriptionally regulated after stimulation with IL-33. Moreover, expression of the chemokine receptor CXCR4 was increased in CD8^+^ T lymphocytes treated with the selective S1P_4_ receptor agonist CYM50308. We conclude that nutrient deprivation promotes CD8^Low^ T lymphocytes, contributing to an immunosuppressive microenvironment and a poor anti-cancer immune response by limiting cytotoxic effector functions. Our results suggest that S1P_4_ signaling modulation may be a promising target for anti-CXCR4 cancer immunotherapy.

**Graphical Abstract:**
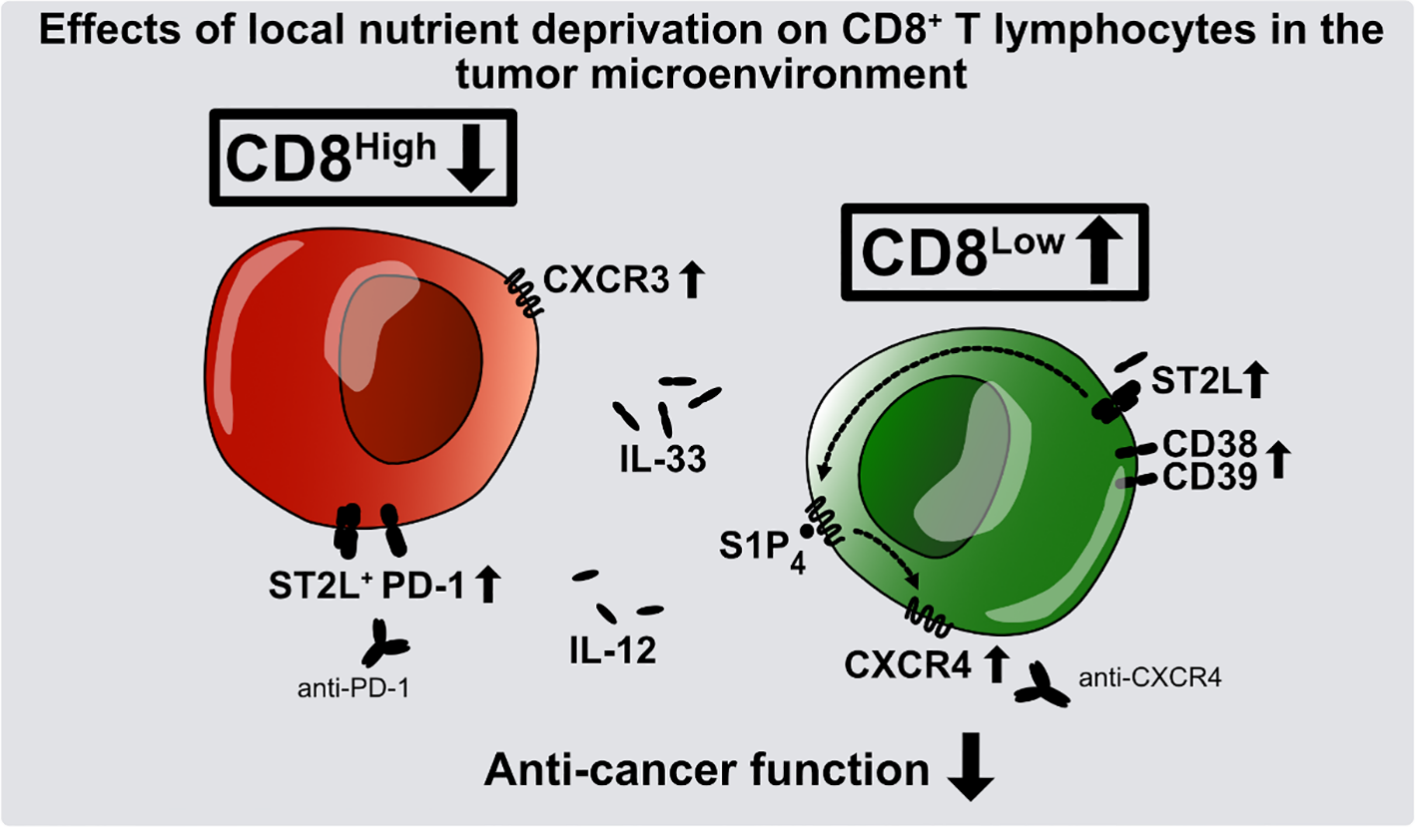


## Introduction

The tumor microenvironment is defined by local nutrient deprivation and an immunosuppressive milieu originating in part from an accumulation of regulatory T lymphocytes (T_reg_) within the tumor tissue (1, 2). Since nutrient deprivation is an important metabolic stressor of immune cells, *in vitro* serum starvation has been proposed as a tool for immune regulation (3) and a useful model for the nutrient-deprived tumor microenvironment (4–6). Tumor-infiltrating immune cells including CD8^+^ T lymphocytes have been described to be key effector cells for the tumor-eliminating cytotoxic immune response. However, since inefficient T cell responses can cause tumors to develop immune resistance, potent cancer immunotherapies aim to enhance T cell recruitment and cytotoxic effector functions within the tumor microenvironment. Immune checkpoint inhibitors target inhibitory receptors such as the programmed cell death protein 1 (PD-1), promoting immune cell-mediated tumor cell elimination (7, 8). Additionally, ectonucleotidases such as CD38 and CD39 produce adenosine, a mechanism required for immunosuppression by T_reg_ (9). Such immunosuppressive purinergic mechanisms within the tumor microenvironment are currently targeted by drugs seeking to restore functional effector anti-tumor immunity, administered in combination with immune checkpoint inhibitors (10). Nevertheless, mechanisms that interfere with the immune response within the tumor microenvironment remain in focus in the pursuit of additional therapeutic targets.

T cell recruitment into tumors is essential for efficient immunotherapy. Chemokine receptors control the cell positioning and migratory potential of the lymphocytes by regulating chemotaxis, mediating pathological anti-cancer immune responses [for review see (11)]. The inflammatory chemokine receptor CXCR3 (CD183) is expressed on activated, pro-inflammatory T lymphocytes (12) and evidentially plays a decisive role in T cell effector migration into tumors (13). Moreover, tumors highly express CXCL12 (SDF1α), the ligand for CXCR4 (CD184), a homeostatic chemokine receptor found to be mostly involved in attracting immune cells within the tumor tissue, contributing to an immunosuppressive milieu (14). The CXCR4 inhibitor Plerixafor (AMD3100) was approved in 2009 for the treatment of patients with multiple myeloma and non-Hodgkin lymphoma (15). Recent advances in CXCR4 antagonist development seek to exploit its potential anti-cancer effects in combination with immune checkpoint inhibitor therapy (16). Alongside chemokines, sphingosine-1-phosphate (S1P) is an important regulator of lymphocyte trafficking, especially during T cell development and differentiation. As a biologically active sphingolipid, it can signal through one of five different G-protein coupled receptors (S1P_1_-S1P_5_) (17). S1P_1_, S1P_2_ and S1P_4_ have been described to be expressed on T lymphocytes (18). S1P_4_ is largely expressed by immune cells. However, its role in T lymphocyte migration and during lymphocyte differentiation is less well understood. In the literature, a minor role of S1P_4_ in cell migration rather than in lymphocyte differentiation (e.g., proliferation, cytokine production) is presumed (19). A recent report highlights a pro-tumorigenic role of S1P_4_ in CD8^+^ T lymphocytes, showing that *S1P_4_* ablation in mice correlates with reduced tumor proliferation and higher CD8^+^ T cell expansion (20).

Cytokines shape the phenotype of immune cells and thus have an impact on the adaptive immune response. Interleukin-33 (IL)-33, which belongs to the family of IL-1 cytokines, was initially described as a driver of T helper 2 (T_H_2) immune responses (21). IL-33 is abundantly expressed as a full-length but biologically active molecule upon cell damage or stress (e.g., in endothelial and epithelial cells) and is therefore classified as an “alarmin” (22). It exerts its function on immune cells by signaling *via* suppression of tumorigenicity 2 (ST2)L transmembrane receptor (23, 24). Recent evidence showed IL-33 to promote tumorigenesis of intestinal cancer by enhancing the accumulation of ST2L^+^ and FoxP3^+^ T_regs_ within the tumor microenvironment (25, 26). In contrast, CD8^+^ T lymphocyte activation and promotion of the potent effector functions are triggered by the inflammatory cytokine IL-12 (27). Alongside T cell receptor (TCR) signaling and IL-2 stimulation, IL-12 is described as the “third signal” for the expansion of cytotoxic T_c_1 immune cell responses. One focus of our recent report was to understand the role of IL-33 and IL-12 in CD8^+^ T lymphocyte differentiation under nutrient deprivation (28). The function of IL-33 is dependent not only on TCR signaling, but also on the presence of IL-12. The latter, acting *via* IFN-γ signaling, accounts for anti-tumor effects that have been extensively discussed elsewhere. It can nevertheless fail to establish robust antitumor responses of CD8 T lymphocytes (29). Both nutrient deprivation and co-stimulation with IL-33 and IL-12 resulted in low T cell specific CD8 expression, upregulation of ST2L, low cytotoxicity, and induction of the regulatory transcription factor FoxP3 (28). Moreover, clinical studies emphasize the role of downregulated CD8 expression in cancer patients (30–32). Building on these findings, we now sought to further characterize the CD8-low-expression (CD8^Low^) lymphocyte subpopulation. The analysis of transcriptional expression of *S1P_1_* und *S1P_4_* mRNA under IL-33 and IL-12 stimulation encouraged us to extend studies on the role of S1P_4_ to examine regulation of the CXCR4 and CXCR3 chemokine receptors, that are known to have opposed functions with regard to T lymphocyte effector and regulatory function. CD8^Low^ T lymphocytes showed highest responsivity to IL-33, upregulating not only ST2L, but also the regulatory immune cell markers CD38 and CD39. Our study establishes an enhancing role of S1P_4_ on CXCR4 expression in CD8^Low^ T lymphocytes during nutrient deprivation. This strongly suggests beneficial therapeutic effects of CXCR4 antagonism of potentially immunosuppressive CD8^+^ T lymphocytes.

## Material and Methods

### Isolation of Primary Human CD8^+^ T Lymphocytes

Human peripheral blood mononuclear cells (PBMC) were isolated from blood donor-derived buffy coats by density gradient centrifugation with Ficoll-Histopaque 1.077 g/mL density (Sigma-Aldrich, Steinheim, Germany) as separation medium. CD8^+^ T lymphocytes were subsequently purified from PBMC by immunomagnetic negative selection using an EasySep™ Human CD8^+^ T cell kit according to the manufacturer’s recommendations (STEMCELL Technologies, Cologne, Germany). Initial assessment of CD8^+^ T lymphocyte purity from several independent experiments confirmed constantly high purity (>95%) of CD3^+^CD8^+^ T lymphocytes after the purification procedure from human PBMC using flow cytometry (data not shown). The studies on human T lymphocytes were performed with buffy-coats from anonymous healthy blood donors of the blood donation center DRK-Blutspendedienst Baden-Württemberg-Hessen, Institut für Transfusionsmedizin und Immunhaümatologie Frankfurt am Main, Frankfurt, Germany. Prior to blood sampling, all participants were informed in full about all aspects of the study and asked to give written informed consent for use of the samples. According to the institutional ethics committee of the Goethe University Hospital, Frankfurt, Germany, and the local legislation, additional ethical approval was not required, since the cells derived from buffy coats were used anonymously for *in vitro* experiments with no link to personal data of the donors.

### *In Vitro* Cultivation of Primary Cells

After the enrichment from human PBMC, CD8^+^ T lymphocytes were cultivated under serum withdrawal (starvation) or, if otherwise indicated, with 10% autologous and heat-inactivated donor serum in RPMI 1640 + Glutamax supplemented with 50 mM β-mercaptoethanol, 1 mM sodium pyruvate, 100 μg/mL streptomycin, 100 IU/mL penicillin (all from Gibco, Waltham, USA) and 2 nM HEPES (Sigma-Aldrich, Steinheim, Germany). Lymphocytes were seeded at a density of 5 x 10^5^ cells per mL into 12-well plates (Greiner bio-one, Frickenhausen, Germany), if not otherwise stated. CD8^+^ T lymphocytes were treated with the interleukins IL-33 at 20 ng/mL and IL-12p70 (hereafter IL-12) at a concentration of 5 ng/mL after 20 h cultivation. These cytokines were purchased from Peprotech, Hamburg, Germany and dissolved for use in PBS/0.1% BSA. Additional pharmacologic modulation of S1P receptors was achieved by daily treatment during the cultivation of CD8^+^ T lymphocytes. A time schedule for the cultivation of CD8^+^ T lymphocytes is provided within the supplement (**Supplementary Figure 1**). FTY720-phosphate (FTY720-P; Novartis, Basel, Switzerland) was dissolved in dimethyl sulfoxide (DMSO). For use as S1P receptor modulators in cell culture, S1P and FTY720-P were pre-diluted in fatty acid-free PBS/0.1% BSA solution and diluted to a final concentration of 200 nM. The cells were consistently stimulated with the selective S1P_4_ receptor agonist [CYM50308 (33)] and S1P_4_ receptor antagonist [CYM50358 (34)] at 200 nM (Tocris, Bristol, UK).

### Quantitative PCR

RNA from CD8^+^ T lymphocytes was extracted using the Isolate II RNA Micro Kit (Bioline, Heidelberg, Germany) according to the manufacturer’s instructions. RNA was then transcribed into cDNA by reverse transcriptase with the Precision nanoScript Reverse Transcription Kit (Primerdesign, Southampton, UK) using the RT-PCR program (65°C for 5 min, 55°C for 120 min and 75°C for 15 min). To quantify KLF2 (Hs00360439_g), S1P_1_ (Hs00173499_m1) and S1P_4_ (HS02330084_s1) mRNA levels in CD8^+^ T cells, relative gene expression was calculated by normalization to the housekeeping genes GAPDH and RPL13A (Primer Design, Southampton, UK) with the 2^−ΔCt^ method (all probes were obtained from Applied Biosystems, Waltham, USA). Gene expression of *S1P1* on CD8^Low^ and CD8^High^ T lymphocyte subpopulations was analyzed after sorting the cells. For the isolation of RNA from CD8^Low^ and CD8^High^ the RNeasy micro kit (Qiagen, Hilden, Germany) was used according to the manufacturer’s instructions. Subsequent cDNA synthesis was performed using SuperScript™ VILO™ Master Mix (ThermoFisher, MA, USA) according to the manufacturer’s instructions.

### Flow Cytometry

For the characterization of surface molecules on CD8^+^ T lymphocytes, cells harvested from culture plates were stained as a single cell suspension with the following antibodies: anti-CD8-V450 (clone RPA-T8, BD Biosciences, Heidelberg, Germany), anti-CD39-BV510 (clone A1), and anti-PD-1/CD279-APC (clone EH12.2H7, both from Biolegend, San Diego, USA), anti-CD38-APC-eFlour780 (clone HIT2), and anti-CXCR4/CD184-APC (clone 12G5, both from eBiosciences, Waltham, USA), anti-CXCR3-Flourescein (clone 49801), and anti-CXCR7-PE (clone 11G8) (both from R&D Systems, Minneapolis, USA). All antibodies were titrated to determine optimal concentrations. In order to avoid non-specific antibody binding, cells were incubated for 10 min at 4°C with 0.1% PBS/FCS containing human F_c_ block (BD Pharmingen, Heidelberg, Germany). Cells were fixed in 2% PFA FACS buffer (PBS with 1% FBS and 0.1% NaN_3_). Samples were acquired with a Canto II flow cytometer (BD Biosciences, Heidelberg, Germany) and analyzed using FlowJo software V7.6.5 (Treestar, Ashland, USA). For gating, fluorescence minus one (FMO) controls and unstained controls were used. The instrument calibration was regularly controlled using Cytometer Setup and Tracking beads (BD Biosciences, Heidelberg, Germany). The general strategy for gating of CD8^Low^ and CD8^High^ subpopulations during analysis of flow cytometry data is provided in the supplements (**Supplementary Figure 2**).

### Suppression Assay

The capacity of starved regulatory CD8^+^ T lymphocytes to suppress the proliferation of responder T cells was assessed using a transwell *in vitro* suppression assay. To this end, human PBMC were labelled with violet cell trace (10 µM, ThermoFisher, MA, USA) according to the manufacturer’s instructions. For the proliferation of labeled responder T cells, PBMC (6 x 10^5^/mL/96-well) were cultivated in 1% autologous donor serum and stimulated with 25 µL/mL ImmunoCult™ Human CD3/CD28/CD2 T Cell Activator (αCD, Stemcell Technologies, Vancouver Canada). In parallel, 2 x 10^5^ purified CD8^+^ T lymphocytes from the same donor were seeded into inserts of transwell cultivation plates (0.4 µm, Corning Costar) and co-stimulated with IL-33 and IL-12 (for details see section 2.2) but initially separated from proliferating responder cells. On day 2, co-culture was started by transferring the insert plate into the 96-well plate with labeled and proliferating T cell responders (lower compartment). The assay was performed with *n* = 3 technical replicates. As a control, 50 ng/mL of rapamycin (LC Laboratories, MA, USA) was added to T cell responders in order to assess the maximal inhibition of proliferation at the beginning of co-culture on day 2. The experimental set-up and time schedule is depicted together with the results in **Figure 3A**. After 4 days of co-culture, proliferation of labeled T cell responders was measured by flow cytometry. Samples were acquired using MACSQuant 10 cell analyzer (Miltenyi Biotech, Bergisch Gladbach, Germany). Modelling of proliferation was done using the analysis tool from FlowJo software V10.7.1 (Treestar, Ashland, USA).

### Cell Migration

For the transwell migration assay, 2.5 x 10^5^ purified CD8^+^ T lymphocytes were seeded in serum-free RPMI 1640 medium into the upper compartment of Boyden chamber (5 µm, Corning Costar), cultivated for 40 h and stimulated with S1P receptor modulators as described above. Inserts were pre-coated for 1 h with 10 µg of fibronectin (Sigma-Aldrich, Steinheim, Germany). Directly before migration of the lymphocytes, the medium within the lower compartment was replaced with fresh medium containing 20 nM of diluted recombinant chemokine CXCL12/SDF1α (Peprotech, Hamburg, Germany) as chemoattractant. CD8^+^ T lymphocytes were then allowed to transmigrate into the lower compartment for 2 h. The absolute number of transmigrated cells was determined using MACSQuant 10 cell analyzer (Miltenyi Biotech, Bergisch Gladbach, Germany). The assay was performed with *n* = 3 technical replicates for each stimulation of individual donor samples. In order to exclude dead cells from the cell count, only DAPI-negative events were considered transmigrated lymphocytes.

### Statistics

Statistical analysis and graphical presentation of data were performed using GraphPad Prism 8 software (La Jolla, CA, USA). If not otherwise stated, all data are presented as means ± SD. Statistical differences between groups were calculated with the Wilcoxon matched pairs signed rank test. Tests for multiple comparisons included the RM one-way ANOVA and Friedman tests. Parametric or non-parametric tests were applied upon testing of normal distribution of data. P-values below 0.05 indicated statistical significance (n.s. for p > 0.05, * for p ≤ 0.05, ** for p < 0.01, *** for p < 0.001 and **** for p < 0.0001).

## Results

### *In Vitro* Nutrient Deprivation Induces CD8^Low^ T Lymphocytes and Affects Transcriptional Levels of S1P_1_ and S1P_4_

**Figure 1:**
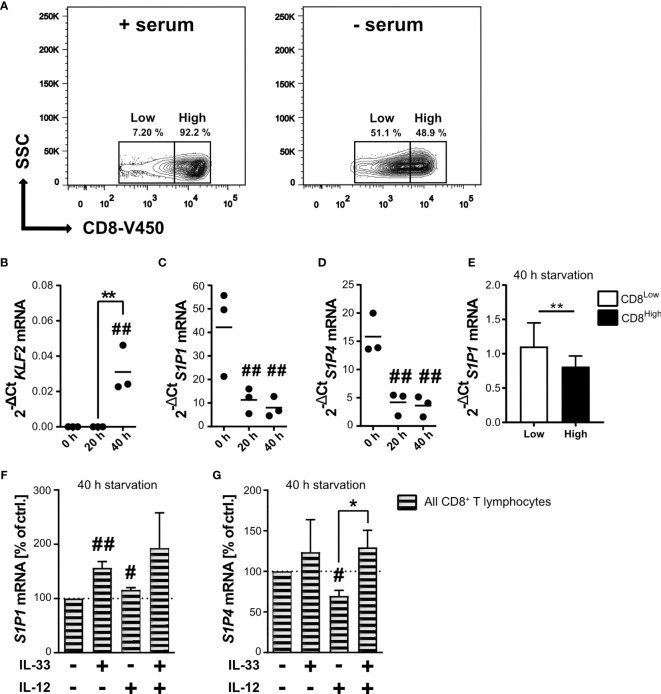
Starvation of CD8^+^ T lymphocytes induces low CD8 expression (CD8^Low^) and IL-33 stimulation regulates the transcriptional levels of *S1P_1_* and *S1P_4_* mRNA. **(A)** CD8^+^ T lymphocytes were purified from human buffy coats, cultivated with 10% autologous donor serum (left) or without serum (right) for 40 h. Representative contour plots show gating of low and high CD8 (V450) expression, which was analyzed by flow cytometry. Total RNA of CD8^+^ T lymphocytes was extracted directly after isolation, and after 20 h or 40 h of cultivation under serum starvation for qRT-PCR analysis of KLF2 **(B)**, S1P_1_ **(C, E, F)**, S1P_4_ **(D, G)**. **(E)** **p < 0.01, for comparisons between CD8^Low^ und CD8^High^ using Wilcoxon matched-pairs signed rank test. Data are obtained from *n* = 9 donors. **(F, G)** Lymphocytes were additionally stimulated with IL-33 (20 ng/mL) and/or IL-12 (5 ng/mL). Data are shown as individual values obtained from *n* = 3 different donors of three independently performed experiments. The horizontal line indicates the mean. Bars show mean ± SD. ^#^p ≤ 0.05, ^##^p < 0.01 for comparisons to the 0 h time point. *p ≤ 0.05, using one-way ANOVA with Tukey´s post-test.

Nutrient restriction within the tumor microenvironment dampens the activity of mTOR, an essential factor for the activity of potentially infiltrating CD8^+^ T lymphocytes. Serum starvation of human CD8^+^ T lymphocytes allowed the differentiation of two subpopulations, the CD8^High^- and CD8^Low^-expression T lymphocytes (**Figure 1A** and **Supplementary Figure 1**). Since the subpopulation of CD8^Low^ increased during *in vitro* nutrient deprivation, we decided to further investigate the specific phenotype of these CD8^Low^ T lymphocytes by determining whether starvation modulates gene expression of the transcription factor *krüppel-like factor 2* (*KLF2)* in CD8^+^ T lymphocytes, eventually resulting in *S1P_1_* mRNA. We found that *KLF2* mRNA expression was significantly induced after 40 h starvation (**Figure 1B**), whereas levels of *S1P_1_* mRNA significantly decreased even after only 20 h nutrient deprivation (**Figure 1C**). Since other studies showed that *KLF2* induction is followed by upregulation of *S1P_1_* (35), we asked whether this initial opposing observation could be explained when analyzing *S1P_1_* mRNA in CD8^Low^ and CD8^High^ subsets separately. As shown before, *KLF2* mRNA and CD8^Low^ were simultaneously induced upon 40 h of starvation, and indeed, we determined an increased transcription of *S1P_1_* limited to CD8^Low^ lymphocytes (**Figure 1E**). While the role of S1P_1_ in lymph node egress has previously been extensively discussed, the function of S1P_4_ remains largely unknown. Therefore, we examined and compared the transcription of *S1P_1_* and *S1P_4_* (**Figure 1D**). Serum starvation had a major impact on mRNA expression levels of *S1P_4_*. Prolonged starvation of 40 h significantly decreased overall *S1P_4_* mRNA expression in all CD8^+^ T lymphocytes, an effect comparable to that observed for *S1P_1_* mRNA expression levels. Thus, nutrient deprivation *in vitro*, which could be modeled by mTOR inhibition, appeared to strongly affect the expression of S1P receptors 1 and 4. Despite the presence of IL-12 as a factor in driving cytotoxic antitumor response, IL-33 and IL-12 together can co-induce a T_reg_-like phenotype in starved CD8^+^ T lymphocytes, as we recently reported (28). To this end, serum starved CD8^+^ T lymphocytes were stimulated with IL-33 and IL-12 after 20 h nutrient deprivation, prior to gene expression analysis of *S1P_1_* and *S1P_4_* after 40 h. Interestingly, gene expression levels of *S1P_1_* were significantly increased after stimulation with IL-33. On the other hand, co-stimulation with IL-33 and IL-12, which has previously been associated with differentiation of a T_reg_-like phenotype, reverted the increase in *S1P_1_* mRNA expression (**Figure 1F**). Since S1P_4_ is the least well-explored S1P receptor, no stimuli for the expression of *S1P_4_* have yet been described. We next aimed to discover whether transcriptional expression of S1P_4_ was upregulated when subjected to nutrient deprivation and cytokine stimulation. Therefore, we serum starved CD8^+^ T lymphocytes and applied the combination of IL-33 and IL-12 – known to trigger a T_reg_ differentiation program in CD8^+^ T lymphocytes during nutrient deprivation (28) – to investigate *S1P_4_* mRNA expression levels (**Figure 1G**). Stimulation with the pro-inflammatory cytokine IL-12 also caused a slight but significant decrease of the gene expression of *S1P_4,_* similar to that observed for *S1P_1_*. Co-stimulation of serum starved CD8^+^ T lymphocytes with IL-33 and IL-12 significantly re-established mRNA expression of *S1P_4_*, which had been downregulated by IL-12 treatment. Hence, we observed, to our knowledge for the first time, that *S1P_4_* gene expression was re-established in CD8^+^ T lymphocytes by IL-33 during nutrient deprivation.

### ST2L Marks CD8^+^ T Lymphocyte Subsets With Increased Expression of CD38, CD39 and PD-1

**Figure 2:**
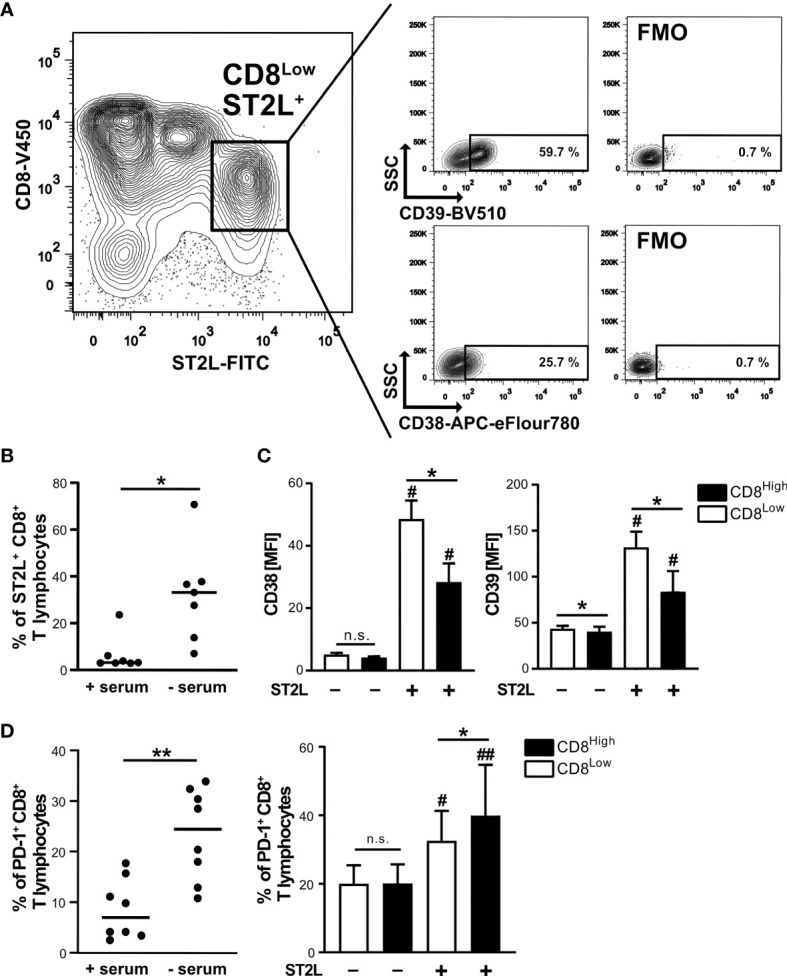
High ST2L expression correlates with enhanced CD38, CD39 and PD-1 surface expression on CD8^+^ T lymphocyte subsets during nutrient deprivation. **(A)** CD38 and CD39 expression on CD8^Low^ ST2L^+^ T lymphocytes during nutrient deprivation. CD8^+^ T lymphocytes were purified from human PBMC and cultivated for 40 h under serum withdrawal. Representative FACS plots show the percentages of CD39^+^ (upper plots) or CD38^+^ (lower plots) CD8^Low^ ST2L^+^ T lymphocytes. FMO: Fluorescence minus one stainings (without either CD39-BV510 or CD38-APC-eFlour780). **(B)** ST2L expression on all gated CD8^+^ T lymphocytes during serum starvation (- serum) compared to a cultivation control (+ serum). The horizontal line indicates the median calculated from *n* = 8 individual donors from three independently performed experiments. **(C)** Mean fluorescence intensities (MFI) of CD38 (left) and CD39 (right) after gating of CD8^High^ (black bars), CD8^Low^ (white bars) and ST2L-positive (+) or ST2L-negative (–) T lymphocytes. Data are means ± SD from *n* = 6 different donors from three independently performed experiments. **(D)** Percentages of PD-1^+^ CD8^+^ T lymphocytes after 40 h cultivation with either no serum or 10% autologous donor serum (left graph) and on predefined CD8^+^ T lymphocyte subpopulations during starvation (CD8^Low^ white bars, CD8^High^ black bars). The horizontal line indicates the mean, bars represent means ± SD. Flow cytometry data was obtained from *n* = 8 different donors from three independently performed experiments. **(C, D)** ^#^p ≤ 0.05, ^##^p < 0.01 for multiple comparisons of ST2L^+^ and ST2L^-^ subpopulations using Friedman test with Dunn´s post-test, *p ≤ 0.05, **p < 0.01, comparisons between groups using Wilcoxon matched-pairs signed rank test.

**Figure 3:**
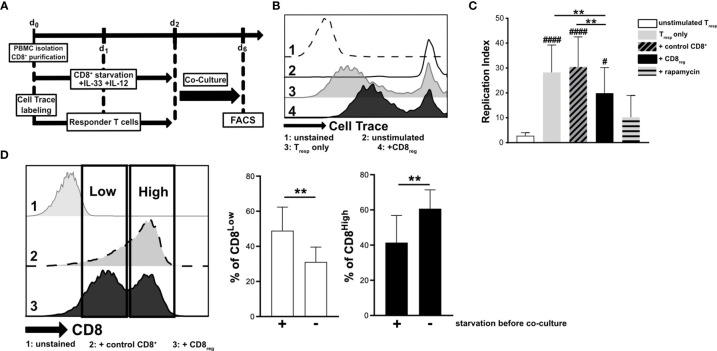
Starved regulatory CD8^+^ T lymphocytes suppress the proliferation of donor-specific responder T cells during co-culture *in vitro*. **(A)** Experimental timeline for transwell *in vitro* suppression with regulatory CD8^+^ T lymphocytes (CD8_reg_) and violet cell trace labeled responder T lymphocytes (T_resp_). For the differentiation of CD8^+^ T lymphocytes and proliferation of labeled T_resp_ using a TCR stimulation cocktail (αCD3/28/2), cells were separately cultivated for 2 days. Co-cultivation of both lymphocyte populations started on day 2 of culture for 4 days before analyzing the proliferation of T_resp_ using flow cytometry. **(B)** Exemplary histograms showing the proliferation of T_resp_ under different conditions after co-culture. 1: unstained control 2: unstimulated T_resp_ control, 3: T_resp_ proliferation alone, 4: Co-Culture with regulatory CD8^+^ T lymphocytes. **(C)** Proliferation of T_resp_ was quantified using the proliferation modelling from FlowJo software. The replication index indicates the fold-expansion dividing cells (expansion capability). **(D)** Representative histograms of CD8^+^ T lymphocytes showing CD8^Low^ (Low) and CD8^High^ (High) subpopulations after co-culture (day 6). 1: unstained control, 2: co-culture with CD8^+^ T cells with no starvation beforehand, 3: co-culture with regulatory CD8^+^ T cells (starved, +IL-33, +IL-12). **(B–D)** All data are obtained from *n* = 9 donors of three independently performed experiments. # for comparisons to the unstimulated control **^#^**p ≤ 0.05, **^####^**p < 0.0001, using Friedman test with Dunn´s post-test. **p < 0.01, using Wilcoxon matched pairs signed rank test.

Since cytokine stimulation of CD8^+^ T lymphocytes impacted transcriptional regulation of the S1P receptors 1 and 4, we aimed to further define the responsiveness of distinct CD8^+^ T lymphocyte subpopulations to IL-33 during nutrient deprivation. To this end, well-defined human CD8^+^ T lymphocyte subpopulations (CD8^Low^ and CD8^High^) were analyzed with regard to ST2L expression (**Figure 2**). CD8^Low^ T lymphocytes clearly showed high ST2L expression (CD8^Low^ ST2L^+^ T lymphocytes) (**Figure 2A**). Of note, overall ST2L expression was lower on CD8^High^ compared to CD8^Low^ T lymphocytes. Accordingly, ST2L expression was increased on CD8^+^ T lymphocytes during starvation compared to serum control, while CD8^Low^ T lymphocytes were not induced (**Figure 2B**). Next, we focused on the potential role of IL-33-responsive CD8^Low^ and CD8^High^ T lymphocytes during nutrient deprivation by analyzing cell surface markers such as the adenosine-converting ectoenzymes CD38 and CD39, which have been proposed as targets for cancer immunotherapy. Having observed that nutrient deprivation induced a subpopulation of CD8^Low^ T lymphocytes, we sought to determine whether these cells express CD38 and CD39. Interestingly, CD8^Low^ ST2L^+^ T lymphocytes exhibited increased CD38 and CD39 surface protein expression compared to CD8^High^ and ST2L-negative CD8 T lymphocyte subpopulations (**Figure 2C**). In general, we observed a higher expression of CD39 compared to CD38. Along with the immunosuppressive adenosine-converting enzymes CD38 and CD39, we also analyzed the expression of PD-1 as an additional important regulator of tumor immune escape. In line with our previous findings, nutrient deprivation conferred higher PD-1 expression on CD8^+^ T lymphocytes compared to serum control (**Figure 2D**, left). On analyzing PD-1 expression on CD8^Low^ and CD8^High^ subpopulations, we found an increase of PD-1 expression by ST2L^+^ cells, especially by CD8^High^ T lymphocytes (**Figure 2D**, right). Taken together, CD8^Low^ ST2L^+^ T lymphocytes exhibited higher protein expression of CD38 and CD39, whereas PD-1 expression dominated on CD8^High^ ST2L^+^ T lymphocytes, potentially limiting the cytotoxic effector functions of CD8^+^ T lymphocytes and thereby presumably promoting a tumor-supporting milieu. Having observed that cytokine stimulation with IL-33 and IL-12 resulted in regulatory CD8^+^ T lymphocyte differentiation during *in vitro* starvation, we decided to perform a functional assessment of cultured cells using a suppression assay. In order to assess the suppressive capacity of regulatory CD8^+^ T lymphocytes, we co-cultured cells with labeled responder T cells from the same donor (**Figure 3A**). Our data indicates that stimulation of starved CD8^+^ T lymphocytes with IL-33 and IL-12 and subsequent co-culture with responder T cells negatively affected the proliferation of responder T cells (**Figures 3B, C**). In addition, we were able to confirm the induction of CD8^Low^ T lymphocytes after co-culture in contrast to the serum control, where the CD8^High^ T lymphocyte subpopulation dominated (**Figure 3D**). These data further support the concept that, under nutrient-deprived conditions, regulatory CD8^Low^ T lymphocytes have the potential to indirectly suppress the effector function of responder T lymphocytes.

### Differential Expression of Homeostatic CXCR4 and the Inflammatory Chemokine Receptor CXCR3 by CD8^Low^ and CD8^High^ Subpopulations

**Figure 4:**
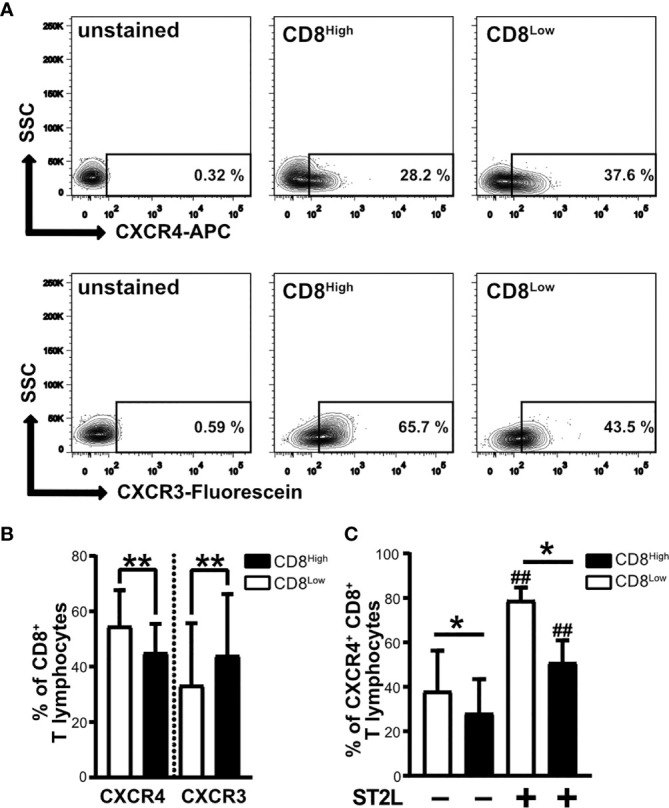
CXCR4 chemokine receptor expression is higher on CD8^Low^ ST2L^+^ T lymphocytes, whereas CXCR3 dominates on CD8^High^ T lymphocytes. Surface protein expression of CXCR4 and CXCR3 on CD8^Low^ and CD8^High^ subpopulations. CD8^+^ T lymphocytes were purified from human PBMC and cultivated for 40 h without serum. CD8^+^ T lymphocyte populations were gated as CD8^High^ (black bars) or CD8^Low^ (white bars) and the expression of CXCR4 (APC), CXCR3 (Fluorescein) or ST2L (FITC) was analyzed by flow cytometry. **(A)** Representative contour plots comparing the percentages of CXCR4^+^ and CXCR3^+^ of CD8^Low^ and CD8^High^ T lymphocyte subpopulations, respectively. **(B)** Data are presented as mean ± SD from *n* = 10 donors of at least three independently performed experiments. **p < 0.01, using Wilcoxon matched pairs signed rank test. **(C)** Data are presented as mean ± SD from *n* = 7 donors of three independently performed experiments. ##p < 0.01 for comparisons of ST2L^+^ and ST2L^–^ subpopulations using Friedman test with Dunn’s post-test, *p ≤ 0.05, comparisons between groups using Wilcoxon matched-pairs signed rank test.

CD8^Low^ T lymphocytes that developed during serum deprivation were characterized by high responsivity to IL-33, showing upregulation of ST2L as well as enhanced expression of CD38 and CD39 as markers of immunosuppression. In contrast, we observed particularly high expression of PD-1 by CD8^High^ ST2L^+^ T lymphocytes. Another denominator of the pro- or anti-carcinogenic role of T lymphocyte subpopulations is their migratory status, which is regulated by different chemokine receptors. For that reason, our interest was drawn to the homeostatic chemokine receptor CXCR4, whose high expression levels were linked to an immunosuppressive phenotype of lymphocytes. Moreover, we wanted to see whether CD8^Low^ and CD8^High^ T lymphocytes express the inflammatory chemokine receptor CXCR3. We found a generally higher frequency of CXCR4^+^ CD8^+^ T lymphocytes within the CD8^Low^ subpopulation, whereas the percentage of CXCR3^+^ CD8^+^ T lymphocytes was significantly higher in the CD8^High^ subpopulation (**Figures 4A, B**). High CXCR4 expression was therefore predominantly observed on the cell surface of CD8^Low^ ST2L^+^ T lymphocytes (**Figure 4C**). These data not only emphasize the differentiating role of nutrient deprivation within a tumor microenvironment, but also support the finding that CD8^Low^ T lymphocytes tend to have a regulatory immune cell phenotype. In contrast to CD8^Low^, CD8^High^ represent typical cytotoxic T cell effectors that are partly inhibited during nutrient deprivation, as indicated by the correlation of PD-1 expression and ST2L within this subpopulation.

### S1P_4_ Receptor Signaling Induces Expression of the Chemokine Receptor CXCR4 by CD8^+^ T Lymphocytes During Nutrient Deprivation

**Figure 5:**
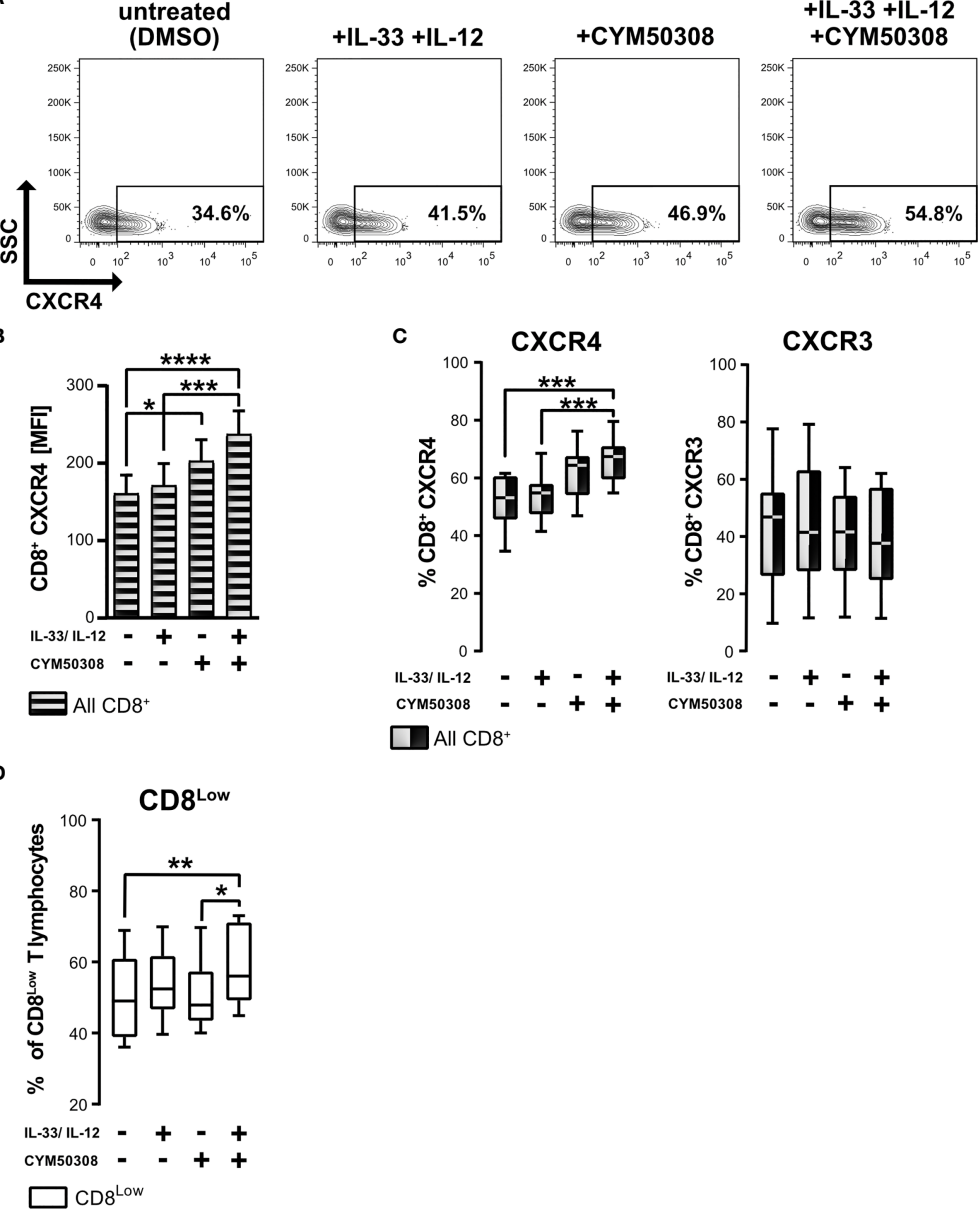
S1P_4_ receptor agonist and cytokine stimulation induces the expression of chemokine receptor CXCR4 and promotes CD8^Low^ T lymphocytes during nutrient deprivation. CD8^+^ T lymphocytes were purified from human buffy coats, cultivated under serum withdrawal for 40 h. Primary cells were stimulated with IL-33 (20 ng/mL) and/or IL-12 (5 ng/mL) and treated daily with the selective S1P_4_ receptor agonist (CYM50308, 200 nM). CD8^+^ T Lymphocytes were stained for CD8 (V450), and stained for the analysis of chemokine receptors surface protein expression with anti-CXCR4 (APC) and anti-CXCR3 (Fluorescein). **(A)** Representative FACS contour plots show the gating of CXCR4^+^ CD8^+^ T lymphocytes. **(B)** CXCR4 mean fluorescence intensity (MFI) of all CD8^+^ T lymphocytes (shaded bars). Data are presented as mean ± SEM. **(C)** Comparison of CXCR4^+^ (left) and CXCR3^+^ (right) CD8^+^ T lymphocytes using the different stimulations. **(D)** Percentage of CD8^Low^ T lymphocytes. **(A–D)** Data obtained from *n* = 11 donors of at least three independently performed experiments. White box plots indicate gated CD8^Low^ T lymphocytes for analysis, whereas black-white plots display data of all CD8^+^ T lymphocytes. The box plots mark the 5^th^ percentile, the median and 95^th^ percentile. *p ≤ 0.05, **p < 0.01, ***p < 0.001, ****p < 0.0001, using Friedman test with Dunn´s post-test **(B, C)** or using Wilcoxon matched pairs signed rank test **(D)**.

Since *S1P_4_* mRNA was expressed in CD8^+^ T lymphocytes under stimulation with IL-33, and despite the presence of IL-12 during nutrient deprivation, we considered whether S1P_4_ might contribute to the regulatory activity and migratory status of human CD8^+^ T lymphocytes. Besides controlling leukocyte trafficking, chemokine receptors have also been shown to perform non-trafficking functions, for example in lymphocyte differentiation (36), affecting tumor immunity. Having determined that CXCR4 expression was high on CD8^Low^ ST2L^+^ regulatory T lymphocytes whereas CXCR3 was dominant on CD8^High^ T lymphocytes, we investigated whether IL-33 and IL-12 stimulation in combination with a pharmacological treatment using a selective S1P_4_ receptor agonist (CYM50308) might alter the expression of the homeostatic chemokine receptor CXCR4 and the inflammatory chemokine receptor CXCR3 (**Figure 5**). Daily treatment with the S1P_4_ agonist was integrated into our existing cultivation protocol of 40 h serum starvation and cytokine stimulation after 20 h. We found that the S1P_4_ receptor agonist, when combined with IL-33 and IL-12 stimulation, induced CXCR4 expression on nutrient-deprived CD8^+^ T lymphocytes (**Figure 5A**). Likewise, when comparing the mean fluorescence intensities (MFI) of CXCR4 on CD8^+^ T lymphocytes, we saw an accumulative effect of cytokine stimulation (IL-33 and IL-12) and S1P_4_ receptor agonist treatment (**Figure 5B**). Of note, S1P_4_ receptor agonist-dependent induction was limited to CXCR4 expression, failing to affect the CXCR3 expression predominant on CD8^High^ T lymphocytes (**Figure 5C**). Moreover, we observed increased proliferation of CD8^Low^ T lymphocytes after cytokine co-stimulation in combination with S1P_4_ receptor agonist treatment (**Figure 5D**). The S1P_4_ receptor agonist was seen to have a dose dependent effect on CXCR4 expression (**Supplementary Figure 3**). Thus, the effects of the S1P_4_ receptor agonist seemed to be restricted to the CD8^Low^ subpopulation. Since S1P_4_ signaling acts as a tumor-promoting factor *via* the proliferation of CD8^+^ T lymphocytes (20), these findings would endorse a potentially tumor-supportive role of CD8^Low^ ST2L^+^ regulatory T lymphocytes, as observed during *in vitro* nutrient deprivation.

### The S1P Receptor Modulator FTY720-P, but Not the S1P_4_ Receptor Antagonist, Increased CXCR4 Expression by CD8^+^ T Lymphocytes

**Figure 6:**
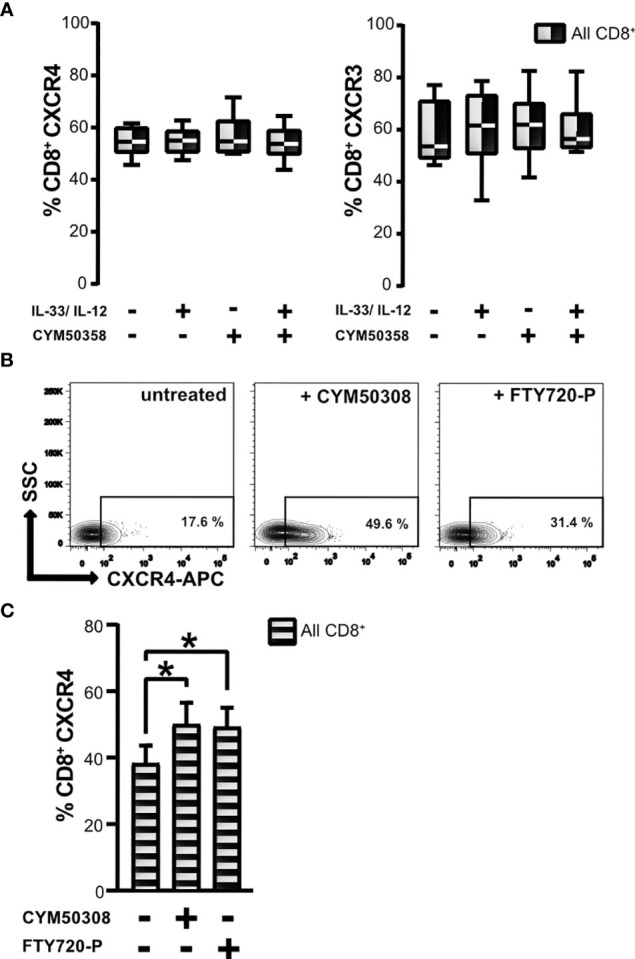
Effects of S1P_4_ receptor antagonist and FTY720-P on chemokine receptor expression. **(A)** CD8^+^ T lymphocytes were stimulated with IL-33 (20 ng/mL) in combination with IL-12 (5 ng/mL), and treated with the selective S1P_4_ receptor antagonist CYM50358 (200 nM). Percentages of CXCR4^+^ (left) and CXCR3^+^ CD8^+^ T lymphocytes (right) are displayed as box plots which mark the 5^th^ percentile, the median and 95^th^ percentile. Data are obtained from *n* = 6 individual donors of three independent experiments. All comparisons showed no significance, using Friedman test with Dunn´s post-test. **(B)** Exemplary contour plots of FACS analysis for daily treatment of CD8^+^ T lymphocytes using either the selective S1P_4_ receptor agonist (200 nM) or S1P receptor modulator FTY720-P (200 nM) compared to untreated control. **(C)** Frequencies of all CXCR4^+^ CD8^+^ T lymphocytes (shaded bars) after treatment with S1P_4_ agonist (CYM50308) or fingolimod-phosphate (FTY720-P) compared to untreated control. Data are presented as mean ± SD from *n* = 8 donors of at least three independently performed experiments. *p ≤ 0.05, using Wilcoxon matched pairs signed rank test.

The S1P_4_ receptor agonist treatment effected an upregulation of CXCR4 surface expression, which had previously been found to be linked to the subpopulation of CD8^Low^ ST2L^+^ T lymphocytes. This led us to ask whether a selective S1P_4_ receptor antagonist might show differential effects on the two different chemokine receptors of CD8^+^ T lymphocytes that were examined. Pharmacologic treatment with the selective S1P_4_ receptor antagonist induced no changes in CXCR4 and CXCR3 expression of CD8^+^ T lymphocytes, neither when given alone, nor when combined with IL-33 and IL-12 stimulation (**Figure 6A**). Beyond the selective modulation of the S1P_4_ receptor, we additionally used fingolimod phosphate (FTY720-P), which acts as a S1P receptor agonist on S1P_1_, S1P_3_, S1P_4_ and S1P_5_, but not on S1P_2_ (37). Interestingly, application of the treatment protocol of CD8^+^ T lymphocytes described above resulted in a comparable enhancement of CXCR4 expression (**Figures 6B, C**). We assume that the observed effects of FTY720-P are attributable to its agonistic effects on the S1P_4_ receptor, since the selective S1P_4_ receptor agonist, but not the antagonist, induced expression of CXCR4 on CD8^+^ T lymphocytes, potentially driving an immunosuppressive phenotype of CD8^+^ T lymphocytes.

### CXCL12-Dependent Migration of Nutrient-Deprived CD8^+^ T Lymphocytes Is Not Dependent on CXCR4-Inductive Effects *via* S1P_4_

**Figure 7:**
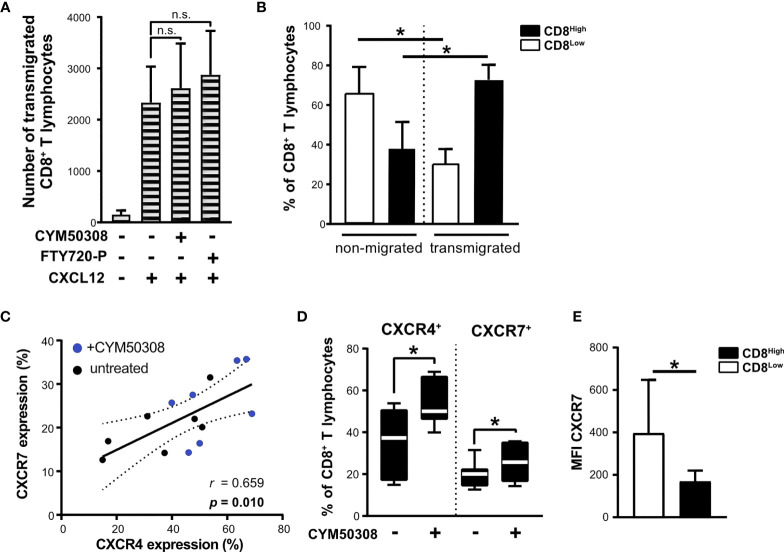
Unchanged migration towards CXCL12 after S1P_4_ stimulation due to lower migratory potential of CD8^Low^ T lymphocytes and co-induction of CXCR7. **(A)** Migration assay for 2 h with 20 nM CXCL12 (SDF1α) within the lower compartment. Absolute count of all transmigrated CD8^+^ T lymphocytes (shaded bars) was quantified using MACSQuant 10. CD8^+^ T lymphocytes were cultivated after purification for 40 h under serum withdrawal within the inserts of Transwell plates. Daily stimulation with either selective S1P_4_ receptor agonist (CYM50308, 200 nM) or S1P receptor modulator FTY720-P (200 nM). Differences in absolute numbers of transmigrated CD8^+^ T lymphocytes between untreated and stimulated samples were not significant (n.s.) using Wilcoxon matched pairs signed rank test. **(B)** Analysis of CD8^Low^ and CD8^High^ T lymphocytes within the upper compartment (non-migrated) and lower compartment (transmigrated cells) after 2 h of migration towards CXCL12. Flow cytometry data are obtained from *n* = 6 donors of three independently performed experiments. **(C)** Positive correlation of CXCR4 (APC) and CXCR7 (PE) protein expression on all CD8^+^ T lymphocytes analyzed by flow cytometry. **(D)** CXCR4 and CXCR7 expression after S1P_4_ receptor agonist stimulation (black-white box plots). The box plot marks the 5^th^ percentile, the median and 95^th^ percentile. **(A–E)** Data are obtained from *n* = 7 donors of three independently performed experiments. Spearman´s correlation (*n* = 14, r = 0.659, *p = 0.01). Dashed lines indicate 95% confidence intervals bands for the regression line. **(E)** Comparison of CXCR7 mean fluorescence intensity (MFI) on CD8^Low^ and CD8^High^, *p ≤ 0.05, using Wilcoxon matched pairs signed rank test.

Nutrient deprivation enhanced the responsiveness of CD8^+^ T lymphocytes to IL-33, which in turn exhibited an induction of *S1P_4_* mRNA expression when combined with IL-12 stimulation. Consequently, S1P_4_ receptor signaling induced the expression of CXCR4, which was linked to a subpopulation of regulatory CD8^Low^ ST2L^+^ T lymphocytes (in contrast to CD8^High^). In order to assess the migratory potential of CD8^+^ T lymphocytes towards CXCL12 (SDF1α), the ligand of CXCR4, we performed a Boyden chamber migration assay. To this end, CD8^+^ T lymphocytes were cultivated under daily stimulation with either S1P_4_ receptor agonist (CYM50308) or FTY720-P within inserts (upper compartment) for 40 h and then allowed to transmigrate along a gradient of CXCL12 into the lower compartment. Neither the S1P_4_ receptor agonist nor FTY720-P was found to significantly enhance CXCL12-dependent transmigration of CD8^+^ T lymphocytes (**Figure 7A**). In this context, we proceeded to compare CD8 expression of transmigrated as well as non-migrated CD8^+^ T lymphocytes, since CXCR4 expression was dominant on CD8^Low^ T lymphocytes. When comparing the frequencies of CD8^High^- and CD8^Low^-expressing T lymphocytes, we clearly found that CD8^High^ exhibited a higher capacity to transmigrate along the gradient of CXCL12, whereas CD8^Low^ generally showed a lower capacity for transmigration (**Figure 7B**). It is known that the expression of CXCR7 (ACKR3), an alternative receptor for CXCL12, might be also relevant to CXCL12-dependent migration. CXCR7 has been reported to bind CXCL12 with high affinity and functions as a scavenging or decoy receptor without exerting chemotactic effects (33). We analyzed in detail whether S1P_4_ receptor agonist treatment of CD8^+^ T lymphocytes affected expression of CXCR7, thus potentially perturbing migratory effects of CXCL12 on CD8^+^ T lymphocytes despite induction of CXCR4. To our surprise, the expression of CXCR4 and CXCR7 on CD8^+^ T lymphocytes showed a positive correlation independent of the treatment of CD8^+^ T lymphocytes (**Figure 7C**). Moreover, the S1P_4_ receptor agonist significantly induced CXCR7 expression, although to lesser extent than CXCR4 expression (**Figure 7D**). Accordingly, CXCR7 expression was higher on CD8^Low^ compared to CD8^High^ T lymphocytes (**Figure 7E**). In summary, our data outline a potential role of two distinct CD8^+^ T lymphocyte subsets that occur during nutrient deprivation and have also been described within a tumor microenvironment. These findings further endorse the possibility that CXCR4, which was predominantly found on CD8^Low^ and is driven by S1P_4_, may affect lymphocyte differentiation rather than being essential for lymphocyte trafficking.

## Discussion

Failure to induce CD8^+^ T lymphocyte effector functions to control cancer cells within the tumor microenvironment is a major limitation for successful cancer immunotherapy. Correspondingly, CXCR4 antagonism, in combination with current anti-cancer immune checkpoint immunotherapy, targeting PD-1, for example, has recently been the subject of discussion and investigation as a therapeutic approach for clinical application (16, 38, 39). Within this study, we investigated the influence of metabolic factors such as nutrient restriction that suggest that this may play a role within a tumor microenvironment and can shape the differentiation and migratory status of CD8^+^ T lymphocytes. During *in vitro* nutrient deprivation of CD8^+^ T lymphocytes, a CD8^Low^-expression subpopulation developed (**Figure 1**), in which we were able to demonstrate high expression of the IL-33 receptor ST2L (**Figure 2**). Some clinical studies report on a low CD8 expression found on tumor-infiltrating T lymphocytes in patients with endometrial carcinoma compared to healthy tissue (30), in patients with lung adenocarcinoma (31) but also low CD8 gene expression in cancer patients with non-response to checkpoint inhibition (32). Further, the downregulation of CD8 expression was connected to lowered antigen sensitivity (40). Interestingly, CD8^Low^ ST2L^+^ T lymphocytes showed upregulation of the adenosine-converting ectoenzymes CD38 and CD39, which have been linked to a suppressive and limited effector function of CD8^+^ T lymphocytes (41, 42). These markers for T cell exhaustion consistently contribute to mechanisms that cause resistance to cancer immunotherapy (43, 44). Our data further support the relevance of alarmin IL-33 for the differentiation of this CD8^Low^ ST2L^+^ lymphocyte subpopulation. When introduced in this study as effector lymphocytes, CD8^High^, a subpopulation inhibited during nutrient deprivation, showed the highest PD-1 expression in the presence of ST2L.

As a starting point for our investigations, we were interested in transcriptional expression of *KLF2*, the transcription factor previously described to drive either S1P_1_ or CCR7 (CD197) protein expression in the context of mTOR-dependent inhibition (45, 46). Due to the lack of reliable antibodies for the detection of S1P receptors on protein level, expression of *S1P_1_* and *S1P_4_* was determined on mRNA level. We found that 40 h starvation induced *KLF2* mRNA, but reduced transcription of *S1P_1_* (**Figure 1**). Expression of the transcription factor *KLF2* has previously been described to be induced during starvation, and, in contrast to our own findings, to promote transcriptional expression of *S1P_1_* (35). However, we were recently able to report that these CD8^+^ T lymphocytes might have a higher potential for CCR7-dependent migration (28). Mechanisms that explain differential transcriptional regulation of *KLF2* and thereby describe a possible link of S1P_1_ and CCR7 are still unknown. Pharmacological inhibition of mTORC1 has been described to promote the expansion and accumulation of T_reg_ (47, 48). Interestingly, the transcription factor *KLF2*, as induced after 40 h *in vitro* starvation, is also required for the generation of regulatory T lymphocytes, and is related to FoxP3 expression (49).

One of our recent reports clearly indicated that CD8^Low^ T lymphocytes play an anti-inflammatory role by connecting CD8^Low^ to cell-lineage specific programs of T_regs_ under co-stimulation of IL-33 and IL-12 (28). In this subsequent investigation, therefore, we analyzed the transcriptional regulation of *S1P_1_* and *S1P_4_* mRNA levels in more detail. IL-33 has been characterized as a functionally ambivalent cytokine that influences the differentiation of immune cells within the tumor microenvironment. Whereas some reports suggest that immunoreactive serum IL-33 is a biomarker for a favorable tumor prognosis (50, 51), other studies outline its pro-tumorigenic role, particularly its effects on anti-tumor immunity. A recent report from Pastille and colleagues stated that IL-33 enhanced the development of regulatory T lymphocytes, thereby promoting intestinal cancer (25). In a murine model, specific depletion of ST2L expression on regulatory T lymphocytes was shown to enhance infiltration of CD8^+^ T lymphocytes and decrease the tumor burden (52). Furthermore, IL-33 deficiency impaired the suppressive function of T_regs_, leading to enhanced beneficial effects of immunotherapy (26). In line with these studies, our data indicate that co-stimulation of CD8^+^ T lymphocytes with IL-33 and IL-12 under nutrient-deprived conditions indirectly suppressed the proliferation of human T cell responders **(** **Figure 3**). Therefore, our data suggest a tumor-supportive role of IL-33 during nutrient deprivation of regulatory CD8^+^ T lymphocytes *in vitro*. However, possible indirect mechanisms of T-cell mediated suppression need to be further described. Dendritic cells are the main producers of IL-12, a modulator of the immune response of CD8^+^ T lymphocytes (53). In contrast to the observed effects of IL-33, IL-12 stimulation of CD8^+^ T lymphocytes alone revealed lower transcriptional mRNA levels of *S1P_4_*, further emphasizing the role of IL-12 as a driver of anti-carcinogenic and cytotoxic immune responses. Although IL-12 is responsible for antitumor responses, we observed predominantly anti-inflammatory effects on CD8^+^ T lymphocytes due to the presence of IL-33 and the serum starvation. In this context, IL-12 was shown to be required for the development of regulatory CD8^+^ T cells and tolerance induction *in vivo*, reflecting the sequential effects of this cytokine (54).

Consequently, IL-33 reversed the effects of IL-12, as shown by the observed significant upregulation of *S1P_4_* mRNA in regulatory-like CD8^+^ T lymphocytes after co-stimulation with IL-33 and IL-12 (**Figure 1**). We therefore considered whether this receptor might fulfill a pro-tumorigenic role associated with the differentiation status and migratory potential of regulatory CD8^+^ T lymphocytes. Recent publications suggest that S1P_4_ supports anti-inflammatory immune responses rather than being significantly involved in lymphocyte migration, as has been described for S1P_1_ (55, 56). S1P_4_, which is mainly found on immune cells, showed no influence on T lymphocyte migration, including chemotaxis to chemokines, but mediates the immunosuppressive effects of S1P (19). In a murine cancer model, *S1P_4_* ablation was shown to reduce the number of T_reg_, but to promote expansion of effector T cells, suggesting that S1P_4_ may play an important role in the development of T_reg_ (20). Interestingly, S1P_4_ promoted expression of the homeostatic chemokine receptor CXCR4, but not of the inflammatory chemokine receptor CXCR3, on CD8^+^ T lymphocytes (**Figure 5**). We also observed more CD8^Low^ T lymphocytes under co-stimulation with IL-33, IL-12 and S1P_4_ receptor agonist treatment, again pointing to a pro-tumorigenic role of CD8^Low^ during nutrient deprivation. This was confirmed by the predominant expression of CXCR4 seen on CD8^Low^ compared to CD8^High^ T lymphocytes, whereas CXCR3 expression was conversely distributed on these two lymphocyte subsets (**Figure 4**). The inflammatory chemokine receptor CXCR3 is highly expressed on activated T cell effectors and has been described to mediate their recruitment to inflammatory sites (57).

CXCL12-CXCR4 chemokine signaling is involved in cancer immune cell trafficking. Our data showed no significantly higher potential of lymphocytes to transmigrate towards CXCL12, especially of CD8^Low^. Although further studies are certainly needed, we anticipate a non-migratory function of CXCR4, reflecting the immunosuppressive differentiation of distinct CD8^+^ST2L^+^ T lymphocyte subpopulations under conditions of metabolic stress. In line with this assumption, many pre-clinical studies report on promising anti-cancer effects of anti-CXCR4 antibodies when applied to target involved immune and cancer cells (58–60). More recent investigations focus on putative therapeutic benefits of CXCR4 antagonism in combination with immunotherapies targeting PD-1 (16). CXCR4 can be characterized as a bone marrow homing receptor (11). The high prevalence of tumor metastasis within the bone marrow can be explained by the presence of high numbers of T_regs_ that create an immunosuppressive milieu (61). This is clearly illustrated by our data describing the prevalence of CXCR4-expressing regulatory CD8^Low^ T lymphocytes during nutrient deprivation.

In summary, not only are tumor-infiltrating cytotoxic CD8^+^ T lymphocytes necessarily exposed to metabolic stress due to local nutrient deprivation, but they are also influenced by immunosuppressive mediators, chemokines and bioactive IL-33 released from necrotic tumor cells. IL-12, which we chose as one driver of IFN-γ mediated antitumor responses, failed to enhance cytotoxic effector functions of CD8^+^ T lymphocytes in the presence of IL-33 during serum starvation. The present study proposes a tumor-promoting role of CD8^Low^ ST2L^+^ regulatory T lymphocytes *via* effects of CXCR4-enhancing S1P_4_ receptor signaling, which is in accordance with current concepts of anti-cancer immunotherapy. Targeting chemokine receptors such as CXCR4, which is predominantly found on regulatory CD8^Low^, may synergistically potentiate blockade therapy targeting PD-1, which we found to be predominantly expressed on CD8^High^ ST2L^+^ T lymphocytes as potential drivers of effective antitumor immunity.

## Data Availability Statement

The original contributions presented in the study are included in the article/**Supplementary Material**. Further inquiries can be directed to the corresponding author.

## Ethics Statement

Ethical approval was not provided for this study on human participants because according to the institutional ethics committee of the Goethe University Hospital, Frankfurt, Germany, and the local legislation, additional ethical approval was not required, since the cells derived from buffy-coats were used anonymously for *in vitro* experiments with no link to personal data of the donors. The patients/participants provided their written informed consent to participate in this study.

## Author Contributions

TB, CD and MH performed the experiments and acquired data. TB wrote the manuscript and designed the figures. HR had the idea, designed and supervised all experiments, checked all data in detail and finalized the manuscript. JP provided material support and basic laboratory equipment. All authors revised the data and reviewed the manuscript for important intellectual content. All authors contributed to the article and approved the submitted version.

## Funding

This work was supported by Else Kröner-Fresenius Foundation (EKFS) and DFG-SFB1039-B03 “signaling by fatty acid derivatives and sphingolipids in health and disease”, both granted to HR.

## Conflict of Interest

The authors declare that the research was conducted in the absence of any commercial or financial relationships that could be construed as a potential conflict of interest.

## Publisher’s Note

All claims expressed in this article are solely those of the authors and do not necessarily represent those of their affiliated organizations, or those of the publisher, the editors and the reviewers. Any product that may be evaluated in this article, or claim that may be made by its manufacturer, is not guaranteed or endorsed by the publisher.

## Abbreviations

FMO, Fluorescence minus one; KLF2, krüppel-like factor 2; MFI, mean fluorescence intensity; PD-1, programmed cell death protein 1; S1P, sphingosine-1-phosphate; S1P_1/4_, sphingosine-1-phosphate receptor 1/4; ST2, suppression of tumorigenicity 2; T_reg_, regulatory T cell.
