# Supplementary material for: Enhanced CXCR4 Expression of Human CD8Low T Lymphocytes Is Driven by S1P4
Source: Front Immunol. 2021 Aug 24;12:668884. doi: 10.3389/fimmu.2021.668884 (PMC8421764; doi:10.3389/fimmu.2021.668884)
Supplement: Supplementary file 1 [file DataSheet_1.docx]

Supplementary Material


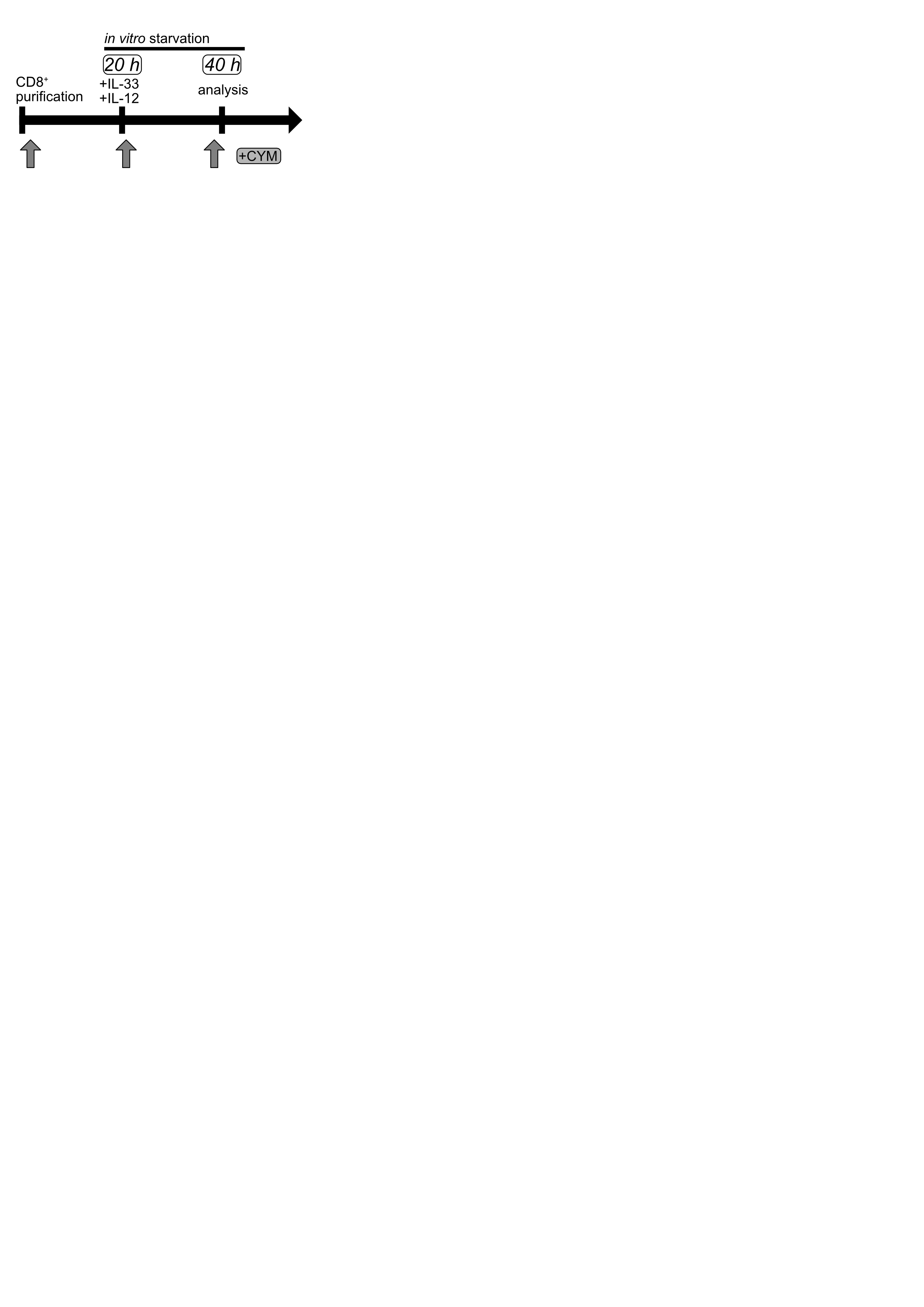


**Supplementary Figure 1: Experimental set-up for the cultivation of human CD8+ T lymphocytes for *in vitro* nutrient deprivation.** For starvation of lymphocytes, cells were cultivated in serum-free T cell medium for 20 h prior to cytokine stimulation with IL-33 (20 ng/mL) and IL-12 (5 ng/mL). Moreover, the selective S1P_4_ receptor agonist (CYM50308, 200 nM) or antagonist (CYM50358, 200 nM) was added daily during the starvation of CD8^+^ T lymphocytes.


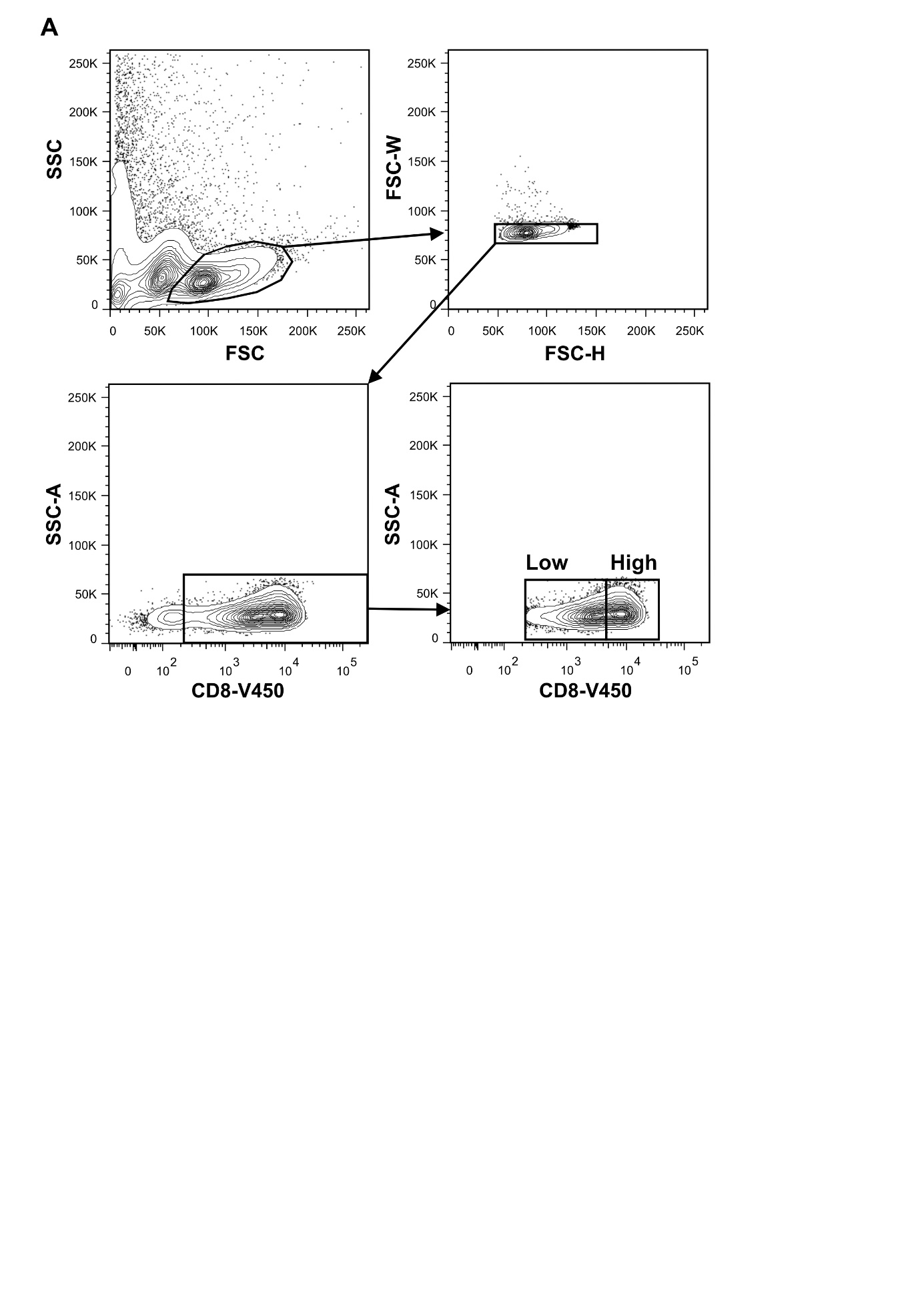


**Supplementary Figure 2: Flow cytometry gating strategy for CD8High and CD8Low subpopulations during nutrient deprivation. (A)** Representative contour plots from purified CD8^+^ T lymphocytes of human buffy coats cultivated for 40 h under serum-free conditions and stained for CD8. Lymphocytes were identified by scatter properties in the FSC x SSC plot and then gated for single cells (FSC-H x FSC-W). For gating of low and high-expressing CD8^+^ T lymphocytes, CD8-negative events were excluded from the FACS analysis. Data were collected with FACSCanto II and analyzed with FlowJo software.


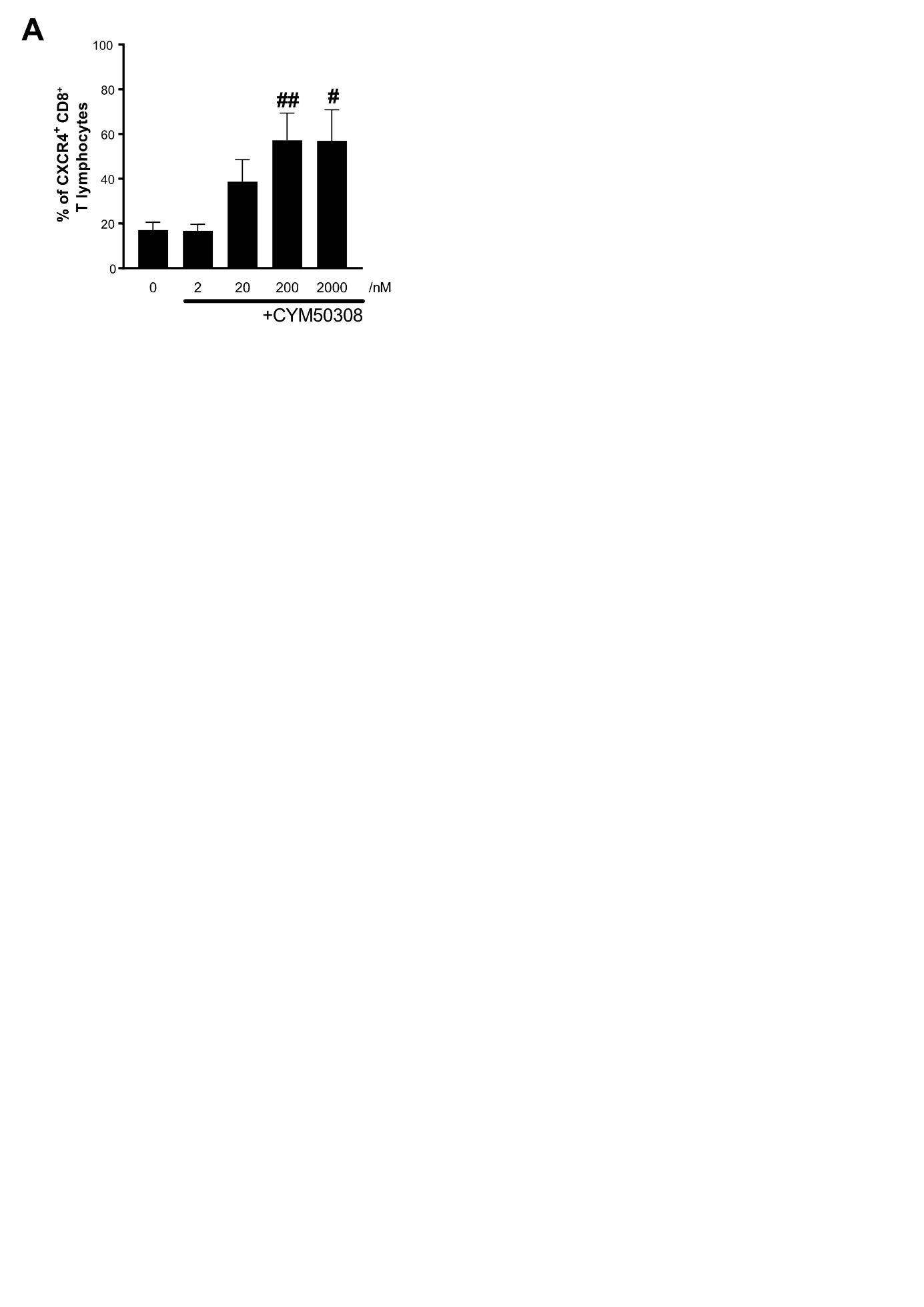


**Supplementary Figure 3: Dose dependent effects of selective S1P_4_ receptor agonist (CYM50308) on the CXCR4 expression of CD8^+^ T lymphocytes during nutrient deprivation.** CD8^+^ T lymphocytes were purified and cultured as previously described. **(A)** Cells were stimulated with either 2, 20, 200 or 200 nM CYM50308 in order to analyze the inductive effects on CXCR4 expression of CD8^+^ T lymphocytes. Data was obtained from *n* = 5 different donors from two independently performed experiments. #p ≤ 0.05, ##p < 0.01 for comparisons with the untreated control using the Friedman test with Dunn’s post-test.
